# The Impact of Alkaline Stress on Plant Growth and Its Alkaline Resistance Mechanisms

**DOI:** 10.3390/ijms252413719

**Published:** 2024-12-23

**Authors:** Shuo Yang, Yiqing Xu, Zhenzhong Tang, Shumei Jin, Shuang Yang

**Affiliations:** College of Life Sciences, Northeast Forestry University, Harbin 150069, China; yangshuo@163.com (S.Y.); xvyiqing2001@126.com (Y.X.); 2024112944@nefu.edu.cn (Z.T.); jinshumei1972@163.com (S.J.)

**Keywords:** alkaline stress, high pH, plant, agricultural production

## Abstract

Alkaline stress can induce significant injury to plants, resulting in a range of negative effects, including ion toxicity, oxidative stress, and damage from high pH values. These stress factors can substantially affect normal plant growth and development, as well as yield and quality loss. To counteract alkaline stress, plants have developed a range of defense strategies, enabling them to adapt and thrive in challenging environments. These defense mechanisms operate at multiple levels such as morphological, physiological, biochemical, and molecular. The continuous advancement of genetic engineering has enabled significant breakthroughs in enhancing plant alkali resistance through human intervention. This research provides a scientific basis for crop production and ecological environment construction, and also promotes the effective development and utilization of saline-alkali lands, improving the sustainability of agricultural production.

## 1. Introduction

Soils that contain higher levels of soluble salts or alkaline substances are known as saline-alkali soils. These soils are more prevalent in regions with improper irrigation practices, poor drainage, or natural conditions that boost salt accumulation [1]. Saline-alkali soils present a worldwide issue, as they not only arrest crop growth but can also jeopardize the diversity, stability, and sustainable development of ecosystems. There are approximately 954 million hectares of saline-alkali land worldwide, and alkaline soil makes up half of the total saline-alkali soil. The annual losses attributed to saline-alkali land are estimated to be around 27.3 billion USD, and the area of such land is expanding at a rate of 1 to 2 million hectares per year [2,3,4]. Consequently, identifying salt-alkaline tolerant plants and exploring their associated mechanisms is at the forefront of global attention aimed at reducing agricultural losses.

Compared with salt stress, alkali stress involves not only osmotic stress and ion toxicity but can also be damaged by high pH values [5]. High pH can inhibit plants’ water-nutrient absorption, enzyme activity, and cause ion toxicity, resulting in suppressed plant growth and even plant death. To counteract alkali stress, plants must not only regulate their intracellular pH to maintain ion balance, but also need to invest considerable material and energy to manage the pH of the rhizosphere microenvironment [6]. Thus, this review aims to uncover the complex physiological mechanisms, biochemical pathways, and molecular adaptations that plants use to withstand the detrimental effects of high soil pH. The focus will be on how plants maintain intracellular pH levels to achieve homeostasis under alkali stress conditions, as well as the broader impacts on plant growth and development. In addition, this article briefly presents current progress and novel methods in plant breeding, genetic engineering, and agricultural practices to enhance plant alkalinity tolerance. By examining current research findings and technological breakthroughs, the aim is to explore management options that enable crops to thrive in alkaline soils. This is important not only for understanding plant adaptation and survival strategies, but also for promoting sustainable agricultural options and informing policy development to mitigate the impacts of alkali stress.

## 2. Effects and Injury Mechanisms of Alkaline Stress on Plants

### 2.1. Impairment of Plant Growth

**Figure 1:**
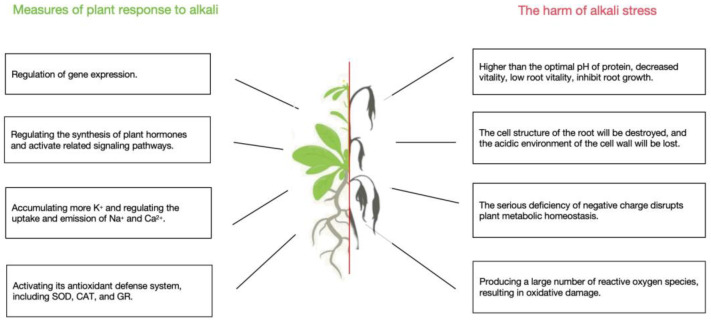
Diagram shows alkali stress tolerance mechanism in plants.

Alkaline stress induces negative impacts on plant growth (Figure 1). During hairy root formation, the initiation site of hairy roots first undergoes cell wall acidification. Once the acidification process concludes, the initiation of hairy roots formation ceases, and the root tip begins to grow [7]. Concurrently with cell wall acidification, the cytoplasmic pH rises from 7.3 to 7.7. However, soil with high pH levels can inhibit the apical growth process of plants, affecting the emergence of root hairs, and subsequently impacting plants’ water-nutrient absorption as well as its normal growth and development [8]. Moreover, when the soil pH exceeds 8.5, auxin levels at the root tip decrease, disrupting the structure of root cells. This loss of the acidic environment in the cell wall leads to the loosening of the cell wall, which in turn hinders cell elongation. Additionally, an increase in ion concentration in the soil can cause osmotic stress for plants, preventing root cells from utilizing the water potential difference between the soil and the plant. This leads to physiological drought in plants [5]. Alkaline stress can also affect chlorophyll content in plant leaves due to a reduction in activity or even inactivation of enzymes related to chlorophyll synthesis under high pH conditions. Furthermore, oxidative stress can also inhibit the synthesis and accumulation of chlorophyll [9,10].Exogenously applied sodium nitroprusside (SNP) can also prevent chlorophyll degradation, restore photosynthetic efficiency, and alleviate the inhibition of photosynthesis in plants under alkali stress [11]. In addition, alkali stress results in a significant accumulation of reactive oxygen species (ROS) in chloroplasts, causing chloroplast swelling, loosening or rupture of grana, and a decrease in photosynthetic capacity [12,13]. SNP can enhance the activity of antioxidant enzymes, promoting the removal of H₂O₂ and helping to protect plants from alkali stress damage [11,14].

Crop growth is related to yield and quality. Major food crops such as rice, wheat, and corn, and major economic crops such as soybeans, cotton, and peanuts highly suffer under high pH stress. Under high pH stress, the germination of rice seeds is significantly inhibited, leading to reduced percentages of brown rice, milled rice, and head rice. Additionally, protein content increases, while the nutritional and taste quality deteriorates [15,16,17]; wheat suffers from poor nutrient absorption and slow growth; corn yield is reduced by 20.0% under moderate saline-alkali soils, and 46.7% under severe cases [18]. Soybean root growth is impeded, leaf chlorosis occurs, and protein synthesis is affected; cotton is sensitive to high pH stress, characterized by slowed growth and decreased fiber quality; peanuts show significant reductions in root system, leaf area, dry weight of various organs, and root-to-shoot ratio [19].

### 2.2. Inhibition of Enzyme Activity

Proteins are essential substances required by plants, playing a crucial role in plant cell and tissue formation, as well as in metabolism. The activity of proteins is greatest at their optimal pH. Many compartments within plant cells, such as the intercellular fluid, vacuoles, and lysosomes, are weakly acidic, typically having pH values between 5.0 and 6.0. Among them, the optimal pH for more than 60 hydrolases ranged from 3.5–5.5. When the pH within plant cells changes, the protonation state of amino acids alters, which affects the activity of various proteins and enzymes to different extents [20,21,22]. For example, the activity of the plant cell plasma membrane H^+^-ATPase is regulated by protein kinases, and the activity of protein kinases is in turn affected by pH [23], and pH change can lead to a decrease in H^+^-ATPases activity with impaired plant growth and development. Additionally, soil enzymes are essential organic components of the soil, actively participating in nutrient cycles and the transformation of nutrient elements [24]. High soil pH can insert more negative impacts on the activity of proteases [25], and when subjected to saline-alkali stress, the activity of soil catalase, protease, urease, and invertase is significantly reduced (Figure 1). The decrease in soil catalase activity disrupts soil ecosystem balance [26]. Furthermore, a reduction in the activity of soil protease, urease, and invertase affects the rate at which insoluble nitrogen, phosphorus, and potassium are converted into soluble compounds. This also decreases the hydrolysis of carbohydrates in the soil, disrupting the nutrient cycle, reducing soil fertility, and ultimately impacting plant growth [27,28].

### 2.3. Alkalinity Induces Ion Toxicity and Osmotic Stress

High external pH values can impact the permeability of root cells to ions, disrupting the cellular ion balance. Alkaline stress causes the precipitation of phosphates and metal ions, resulting in a significant decrease in ion activity and the free concentration of various ions. This results in a reduction of soil water potential, which hampers plant water absorption and leads to osmotic stress, but also several trace elements, such as iron, manganese, zinc, and copper, form insoluble compounds that plants cannot absorb, leading to the inability of plants to turn green and yellow (Figure 1). The metabolism of cations K^+^ and Na^+^ is affected, with an influx of Na^+^ and efflux of K^+^, leading to an increased Na^+^/K^+^ ratio inside cells. Excessive accumulation of Na^+^ can reduce plant absorption of Ca^2+^, thereby destroying the stability of the cell membrane and cell wall structure, and interfering with signal transduction [29]. At the same time, the absorption of anions such as Cl^−^, NO^3−^, and H₂PO₄⁻ is inhibited, leading to a severe deficiency of negative charges and disrupting the metabolic homeostasis of plants [30,31].

Alkaline stress significantly reduces the K⁺/Na⁺, Ca^2^⁺/Na⁺, and Mg^2^⁺/Na⁺ ratios in both the aboveground parts and roots of flax. Additionally, the content of Cl⁻ and NO₃⁻ also decreases, with the reduction in the roots being significantly greater than in the aboveground parts. With the increase in alkaline stress intensity, the content of H₂PO₄⁻ and SO_4_^2−^ in the aboveground parts and roots of flax also decreases [32]. In studies of wild soybean (*Glycine soja*) response to salt-alkaline stress, similar conclusions have been reached. Compared to salt stress, *Glycine soja* seedlings under alkaline stress are unable to accumulate Cl⁻, SO₄^2^⁻, and H₂PO₄⁻ to counterbalance excess Na⁺, which hinders their ability to maintain ion balance and results in more severe root damage [33]. Under saline-alkali stress, Ca^2+^ concentration in the plant cells rapidly increases, activating calcium-dependent protein kinases (CPKs). These kinases, by phosphorylating downstream substrate proteins, initiate osmotic stress signals allowing the plant to react to stress [34]. Furthermore, osmotic stress can also cause secondary damage, such as oxidative stress, which impairs normal plant functioning [35].

### 2.4. Oxidative Stress

Reactive oxygen species are induced by biotic and abiotic stresses. They act as signaling molecules that regulate numerous biological processes in plants. Under high pH stress, plant cells produce and accumulate superoxide anions (O^2−^), hydrogen peroxide (H_2_O_2_), hydroxyl radicals (-OH), and singlet oxygen (^1^O_2_) [36]. ROS can accumulate in chloroplasts, mitochondria, and peroxisomes, disrupting the dynamic balance between ROS generation and degradation (Figure 1). This imbalance leads to lipid peroxidation of cell membranes, increased membrane permeability, and structural damage, ultimately resulting in ion leakage [12,13]. The oxidation of DNA fragments, degradation of RNA, and oxidative damage to lipids and proteins are associated with ROS generation [37].

In plants cells, the degree of lipid peroxidation intensifies with increasing ROS levels. ROS stress leads to the breakdown of fatty acid chains resulting in both cell membrane and cytoplasmic membrane undergoing peroxidation. This consequently induces a lower content of unsaturated fatty acids in the membrane, altering membrane fluidity and disrupting membrane permeability and structure. This further affects normal function of the membrane, which causes ion leakage [38]. Malondialdehyde (MDA), one of the products of membrane lipid peroxidation, reflects the degree of cell membrane damage and the strength of a plant’s resistance to salinity-alkalinity stress. Cytoplasmic membrane damage can result in an increase in MDA content, which further exacerbates damage to the membrane system. Higher MDA content in the alfalfa plant under salinity-alkalinity stress weakens its resistance, and the MDA content is inversely proportional to the salt tolerance of corn [39].

ROS can also directly or indirectly affect proteins in various ways, which can lead to protein modification. Direct modifications include peroxidation of amino acid residues, oxidation of sulfur-containing groups, disulfide bond formation, and glutathionylation; indirect modifications mainly refer to the combination of proteins with fatty acid peroxidation cleavage products to form carbonylated proteins [40,41]. These actions cause damage to proteins, such as the modification of specific amino acids at certain sites, peptide chain breaks, cross-linking and aggregation, changes in charge, and increased sensitivity to proteolytic action. This results in alterations in protein structure, dysfunction, and degradation, all of which can adversely affect normal plant growth [14].

## 3. Plant Alkali Stress Tolerance Mechanism

### 3.1. Changes in Osmotic Regulatory Substances

Alkali stress alters the osmotic pressure within plant cells and causes osmotic stress. Compared with neutral salt stress, high pH induced by alkali stress causes severe and complex injury to plants [42]. With the rapid development of omics technologies, metabolomics has become a popular tool for identifying osmotic regulatory substances.

Proline, as an active oxygen scavenger, plays a significant role in osmotic stress [43,44]. Under saline-alkali stress, the activity of P5CS in alfalfa was increased, resulting in a higher proline content [42], and the proline level in the root system was increased more than 5.72 times under alkaline salt stress in rapeseed [45]. The same accumulation is observed in both linseed (*Linum usitatissimum* L.) [46], groundnut (*Arachis hypogaea* L.) [44] and wolfberry (*Lycium barbarum* L.) [47]. Under abiotic stress, plant nitrogen metabolism is controlled by glutamate formation, which acts as a nitrogen donor in the biosynthesis of transaminases for the production of other amino acids, including proline. Proteomic and metabolite analysis of apple rootstock *Malus halliana* revea significant enrichment in alanine, aspartate, and glutamate metabolism, with increased level of glutamate and arginine [48]. Likewise, rapeseed roots accumulate large amounts of glutamate and glutamine under alkaline stress through the metabolic pathways of alanine, aspartate, and glutamate [45].

Organic acids play a key role in pH regulation. The secretion and formation of organic acids can stabilize the rhizosphere pH and overcome high pH stress-induced injury in plants. Under alkaline salt stress, fatty acids accumulate in large quantities in rapeseed roots [45], and alfalfa roots produce more fatty acids, promoting the β-oxidation process, which produces high energy to combat abiotic stress damage [42]. The TCA cycle is a primary metabolic pathway for energy provision, and its response to saline-alkali stress differs across plants species. In alfalfa, under alkaline salt stress, the expression and activity of TCA cycle-related enzymes rises, leading to organic acid formation in the cycle [42]. In rapeseed, while glycolysis is blocked under alkaline salt stress, the level of organic acids in the TCA cycle rises, due to organic acids content to enhance energy production and stress resistance [45]. Both Broomcorn millet [49] and soybeans (*Glycine soja*) [50] regulate the TCA cycle under alkali stress to increase the organic acid content. These data suggest that plants enhance their stress resistance by modulating the TCA cycle and organic acid metabolism, though the specific mechanisms may vary among species, with a common focus on increasing organic acid formation to combat stress damage.

In plants, flavonoids, important polyphenolic secondary metabolites, play a critical role under stress, scavenging on free radicals and singlet oxygen, which sustains oxidative balance. Under alkali stress, *Glycyrrhiza uralensis* accumulates significant contents of flavonoid compounds, e.g., 2′-hydroxygenistein, apigenin, and 3-*O*-methylquercetin, which greatly contribute to the plant’s resistance to oxidative stress as part of its non-enzymatic antioxidant system [51]. These flavonoid compounds not only build up the antioxidant system but can also help in the plant’s alkali stress tolerance. Similarly, alkali stress upregulates key genes participating in flavonoid compound synthesis in alfalfa (*Medicago sativa* L.) [42]. This further leads to an accumulation of flavonoids, and consequently boosts plants’ antioxidant capacity tolerance to alkali stress.

### 3.2. Role of Plant Endogenous Hormone

Plants can resist adverse stress by increasing the synthesis of endogenous hormones, such as abscisic acid (ABA), indole-3-acetic acid (IAA), and jasmonic acid (JA). IAA, as one of the most important hormones can promote stem and leaf growth as well as roots elongation (Figure 1). Alkaline stress can decrease IAA synthesis and hamper root growth [52]. Exogenous use of IAA can restore root growth and alleviate abiotic stress-induced injury [53]. IAA not only promotes root growth but also enhances plant tolerance to alkalinity stress. For example, IAA treatment can enhance the activity of catalase in cucumber leaves, alleviating sodium alkali stress [54]. Rice treated with exogenous IAA shows increased chlorophyll concentration and photosynthetic rate, as well as enhanced activity of antioxidant enzymes, helping to mitigate the effects of alkaline stress [55]. Under alkaline stress, the abundance of PIN2 in the root tip increases, and the transported IAA activates the activity of plasma membrane H⁺-ATPases, promoting rhizosphere acidification to counteract the damage caused by alkaline stress [56].

**Figure 2:**
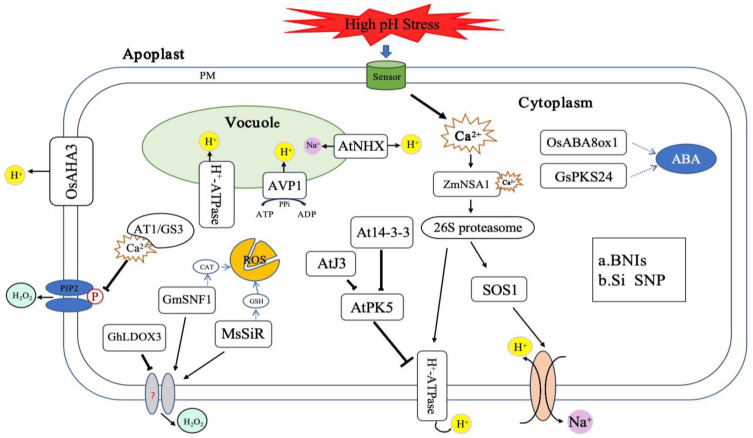
Plant response to high pH stress involves multiple physiological and molecular mechanisms, including the production of reactive oxygen species (ROS), the increase of pH, and the upregulation or downregulation of a series of related genes, which play important roles in the process of plant adaptation to alkaline environments. (a) BNIs secreted by *Leymus chinensis* can release H^+^ against alkali stress; (b) Exogenous Si and SNP help plants under alkali stress to resist ROS and enhance alkaline tolerance.

ABA plays a significant role in plants’ responses to abiotic stress. Under alkaline environments, high levels of endogenous or exogenous ABA can activate antioxidant defense systems to reduce ROS accumulation and counteract oxidative damage [57]. Silencing *OsABA8ox1* raises the endogenous ABA levels in rice, resulting in stronger alkaline tolerance compared to the wild type [58] (Figure 2). Exogenous application of ABA to ABA-deficient mutant (*notabilis*) can promote proline and soluble sugars accumulation and enhanced antioxidant enzyme activity, thus alleviating ROS-induced degradation of chlorophyll and cell membrane damage in *notabilis* leaves [59]. In addition to enhancing the antioxidant system, ABA can alleviate physiological issues caused by salt-alkaline stress by regulating the levels of osmotic substances and chlorophyll. This regulation plays a key role in helping tomato seedlings and alfalfa adapt to salt-alkaline stress [59,60]. An increase in the Na^+^/K^+^ ratio, water loss, and membrane damage caused by alkaline stress are initiated to some extent due to ABA synthesis, thereby reducing the Na^+^/K^+^ ratio. Pre-treatment with ABA can also increase relative water content of aboveground parts of the rice resulting in reduced membrane damage to buds and roots [61]. JA can regulate Na⁺ and K⁺ content, inhibit Na⁺ absorption, enhance the activity of antioxidant enzymes, and improve the elimination of methylglyoxal (MG) toxicity through the GSH-based glyoxalase system, thereby alleviating oxidative damage caused by alkaline stress [62]. Overexpression of the jasmonate ZIM domain (JAZ) family protein GsJAZ2 can protect transgenic *Arabidopsis* plants from alkali stress, enhancing their tolerance to alkaline environments (Figure 2). These studies indicate that various endogenous hormones in plants respond to alkaline stress through multiple pathways, ensuring the plant’s survival under adverse environmental conditions [63].

### 3.3. Antioxidant Defense

Under alkaline stress, higher levels of ROS formation occur in plants and can lead to oxidative injury. The primary method for scavenging reactive oxygen species (ROS) is to enhance the antioxidant defense system. This system includes enzymes such as superoxide dismutase (SOD), catalase (CAT), and peroxidase (POX), as well as non-enzymatic antioxidants like ascorbic acid and glutathione. These antioxidants can neutralize ROS, preventing lipid peroxidation, protein oxidation, and DNA damage, thus stabilizing redox balance, maintaining cellular homeostasis, and enhancing plants’ stress resistance [36] (Figure 1). Pre-treatment with plant hormones such as indole-3-acetic acid (IAA), abscisic acid (ABA), and jasmonic acid (JA) can increase the activity of antioxidant enzymes and reduce oxidative damage. Similarly, exogenous melatonin application can also activate antioxidant enzyme activity and increase related gene expression to reduce ROS accumulation [64]. Anthocyanins, belonging to the flavonoid secondary metabolic products, act as “scavengers” of reactive oxygen to protect plants from abiotic and biotic stresses. Rice pretreated with procyanidins reduced ROS generation with less root injury [65]. Under saline-alkali stress, the overexpression of the anthocyanin synthesis-related gene *GhLDOX3* in *Arabidopsis* leads to plant wilting, playing a negative regulatory role in the mechanism of resisting Na_2_CO_3_ stress. In plant breeding, enhancing anthocyanin synthesis by inhibiting the expression of *GhLDOX3* to clear excessive ROS can thus increase plant alkali tolerance [66] (Figure 2).

*MsSiR* is involved in the synthesis of reduced glutathione (GSH). Overexpression of this gene can lead to an increase in GSH content, cysteine (Cys), and oxidized glutathione (GSSG) in plants, which in turn enhances antioxidant enzymatic activities and alkali stress tolerance [67]. GsMSRB5a, a methionine sulfoxide reductase B5a from *Glycine soja*, can interact with the Ca^2+^/CAM-dependent kinase GsCBRLK. This interaction helps to suppress ROS formation by modifying ROS signaling transduction, biosynthesis, and the expression of inhibitory related genes, thereby enhancing alkali tolerance [68]. The *AT1/GS3* gene suppresses the phosphorylation of water channel protein PIP2, promoting the extrusion of H_2_O_2_ and thus alleviating oxidative damage. Its negative regulatory role in alkali stress tolerance is highly conserved in rice, millet, and maize crops [69] (Figure 2). Silicon enhances the level of carotenoids, protecting chlorophyll from ROS-induced damage by reducing MDA accumulation with an increased antioxidant system [70].

### 3.4. Ion Regulation

For normal plants growth, maintained ion balance under alkaline stress is essential under stressful environments. A wide range of ion channels are utilized by plants for the uptake and extrusion of ions: H^+^-ATPases, H^+^-PPases, and Na^+^/H^+^ antiporters are responsible for extruding excess Na^+^ from the cytoplasm, while K^+^ transporters ensure stable K^+^ levels within the cell and maintain a balanced K^+^ ratio across the cell membrane [35,71,72]. Under alkaline stress conditions, the imbalance of ions prompts plants to activate a series of transporters and ion channels to restore homeostasis [35]. These transporters and ion transport mechanisms are strongly regulated at the transcriptional and post-transcriptional levels [73,74], thus, enabling plants to adjust their ion flux in response to environmental changes, which is essential for normal plant growth.

For instance, the Salt Overly Sensitive (SOS) pathway can extrude excess sodium ions from the cytoplasm, thereby alleviating Na^+^ toxicity while maintaining K^+^ levels imperative for cellular function [75,76]. This pathway exemplifies the complex signaling mechanisms that plants have evolved to sense and respond to ion imbalances, ensuring their survival under alkaline stress conditions. Plants with lower OsRbohD and OsRbohH increase K^+^ and H^+^ extrusion, maintaining the homeostasis of Na^+^/K^+^ and Na^+^/H^+^ [77]. S-Nitrosoglutathione reductase (GSNOR) can enhance the extrusion of Na^+^ and the expression of transport-related genes, activating the Na^+^ detoxification process [78].

Root cells generate a membrane potential difference by extruding H^+^, providing the potential energy needed for nutrient absorption. However, a high pH environmental stress disrupts the H^+^ gradient in the rhizosphere, and H^+^-dependent Na^+^ transport proteins are unable to expel excess Na^+^, exacerbating Na^+^ toxicity [79]. Under saline-alkali treatment, an increase in the cytosolic Ca^2+^ concentration leads to the binding of Ca^2+^ with ZmNSA1, and its subsequent degradation through the 26S proteasome pathway. This increases the transcription level of maize PM-H^+^-ATPases (MHA2 and MHA4) and enhances H^+^-ATPases activity, thereby promoting H^+^ outflow from roots. This enhances the activity of the SOS1 Na^+^/H^+^ antiporter mediating Na^+^ outflow from the roots, ultimately promoting saline-alkali stress tolerance [79] (Figure 1). Once in the rice plasma membrane, functional loss of the H^+^-ATPase *OsAHA3* gene occurs. This exhibits a sensitive phenotype to saline-alkali stress, and overexpression of this gene can improve rice saline-alkali stress tolerance [80] (Figure 2). Protein kinase SOS2-LIKE5 (PKS5) inhibits the activity of H^+^-ATPases by blocking the interaction with 14-3-3 proteins enhancing the *Arabidopsis* pks5 mutant plants’ tolerance to high alkaline stress [81]. On the contrary, Chaperone J3 activates the plasma membrane H^+^-ATPases by inhibiting PKS5, and J3 mutant plants exhibit hypersensitivity to alkaline stress [82].

Vacuoles play a crucial role in regulating pH homeostasis. The *AVP1* gene in *Arabidopsis* encodes the AVP1 proton pump, which can regulate the pH level within the cell sap and act as an auxiliary pump for the V-ATPase large vacuolar pump [83]. Tomato plants transformed with the *AVP1* gene have more developed root systems and stronger salt-alkali tolerance than wild types [84] (Figure 2). The *AtCHX23* gene encodes a Na⁺ (K⁺)/H⁺ antiporter that regulates pH homeostasis in the cytoplasm and chloroplast. This protein, likely functioning as a Na⁺ (K⁺)/H⁺ exchanger on the chloroplast envelope, plays a role in pH homeostasis and chloroplast development in *Arabidopsis thaliana* [85] (Figure 2). Additionally, the *AtNHX1* gene encodes a Na⁺/H^+^ antiporter located in the vacuolar membrane, which helps maintain pH and ion balance in the cytoplasm [86] (Figure 2).

In addition, plants tolerant to ammonium nitrogen can produce rhizosphere secretions through the biological nitrification inhibition (BNI) effect, alleviating nitrogen limitation. *Leymus chinensis* secretes benzoxazinoid derivatives (BNIs) to meet its nutrient requirements while also releasing H⁺ ions to maintain the proton-promoting energy required for nutrient absorption. This process lowers the rhizosphere pH, facilitating better adaptation to alkaline conditions [87] (Figure 2).

### 3.5. Regulation of Gene Expression

Under alkaline stress, certain genes involved in stress perception, signal transduction, and defense mechanisms respond by upregulating or downregulating to promote or inhibit the expression of related proteins. Specific transcription factors also play a crucial role in the expression process of these proteins. These proteins participate in osmotic protection, ion transport, and antioxidant defense. This further enable plants to withstand alkaline conditions (Figure 1). Transcriptome sequencing analysis of *Arabidopsis* under high pH stress has shown that AP2/EREBP, WRKY, NAC, and MYB play key roles in this process [88]. Liu et al. [89] isolated the transcription factor LpNAC5 from lily, and plants overexpressing *LpNAC5* showed enhanced tolerance to alkaline stress. The AP2/ERF family transcription factor GsERF71 from *Glycine soja*, as a DNA-binding protein, can positively regulate *Arabidopsis* alkaline stress tolerance [90]. These transcription factors (TFs) are components of a complex signaling network; they bind to specific promoter regions to regulate the expression of target genes. By integrating external cellular signals and internal responses, they coordinate defense strategies in plants. The alkaline stress response gene family also includes *bZIP*, *C2H2*, *HB*, and *TIFY*, among others. Overexpression of *GsTIFY10a* and *GsTIFY10e* in *Arabidopsis* enhanced plants’ tolerance to alkalinity; *Arabidopsis* knockouts for *AtTIFY10a* and *AtTIFY10b* also confirm the positive role of TIFY10s in the alkaline stress process; transgenic alfalfa with the ectopic expression of *GsTIFY10a* shows increased alkaline tolerance, and these results all indicate that TIFY10 can actively regulate plant responses to alkaline stress [91]. Under alkaline conditions, transgenic soybean overexpressing *GsJAZ2* exhibited significantly better growth than wild type, and overexpression of *GsJAZ2* may increase alkaline tolerance by participating in the JA signaling pathway and accumulating compatible solutes [92] (Figure 2).

Rice SART seedlings adapt to high salinity-alkalinity stress by upregulating the expression of genes associated with the synthesis of cell wall matrix polysaccharides. This results in the accumulation of greater amounts of hemicellulose, pectin, lignin, and suberin in the cell wall. This enhances the mechanical properties and barrier of the cell wall, maintaining the integrity of the cell wall and helping SART seedlings to cope with salt-alkalinity stress in the root system [93]. In the wild soybean (*Glycine soja*), 56 SOS2-like protein kinase (PKS) family genes have been identified. The transcript profiles of the PKS family genes reveal different expression patterns under alkaline stress, and the overexpression of GsPKS24 can enhance the tolerance of *Arabidopsis* and soybean hairy roots to high pH stress. This suggests that GsPKS24 plays a regulatory role in response to NaHCO_3_, pH, and ABA treatments, and its overexpression enhances plant tolerance to pH stress [94] (Figure 2). The serine/threonine kinase SNF1 protein gene is involved in various abiotic stresses in plants. Transgenic *Arabidopsis* and soybean hairy root complex plants overexpressing *GmSNF1*, as well as soybean with *GmSNF1* gene silencing, have demonstrated the salt-alkali tolerance function of the *GmSNF1* gene. *GmSNF1* can be used in genetic engineering to improve plant salt-alkali tolerance [95] (Figure 2). Hexokinase (HXK) proteins catalyze the phosphorylation of hexoses and play a crucial role in signal transduction. Alkaline stress treatment significantly increases the activity and transcription of *GmHXKs*, and the expression level of the B-type isozyme (*GmHXK15*) is strongly related to HXK activity. The overexpression of the *GmHXK15* gene can significantly enhance the resistance of transgenic soybean to alkali stress [96] (Figure 2).

A comprehensive overview of the treatment regimens and diagnostic metrics relevant to recent alkaline stress studies is presented, encapsulating a wide array of parameters (Table 1). These include biomass, oxidative stress, and photosynthetic efficiency, which are essential for understanding the adaptive strategies that plants employ to mitigate the negative impacts of alkaline stress. These data underscore the diverse mechanisms that various plant species utilize to adapt to alkaline environments, revealing distinctions in their physiological and molecular reactions.

## 4. Artificial Intervention Methods for Plant Alkali Tolerance

### 4.1. Traditional Breeding and Selection

For many years, traditional breeding techniques have been employed to enhance plants’ tolerance to various abiotic stresses, including alkali stress. This approach has aided in identifying and crossbreeding numerous plant varieties that naturally exhibit high tolerance to saline-alkali soils. By leveraging genetic diversity within and across plant species, breeders can develop new varieties that exhibit not only enhanced stress tolerance but also improved yields and quality [108]. Marker-assisted selection (MAS) further refines this process, enabling the selection of genes associated with alkali stress tolerance using phenotypic and genotypic information [109]. This method accelerates the breeding process by identifying individuals carrying the desired genes before the manifestation of phenotypic traits.

### 4.2. Genetic Engineering

Due to rapid advancements in molecular biology and biotechnology, genetic engineering is a powerful tool to enhance plants’ tolerance to alkali stress. Using gene editing techniques can precisely introduce, delete, or modify specific genes within the plant genome and thus enhance plants’ ability to attain high pH ambient [110,111]. These targeted genes typically encode stress-protective proteins, regulate ion transport mechanisms, and facilitate the synthesis of osmotic protectants and antioxidants, which are important for plants to withstand alkali stress [110].

Genome-wide association studies (GWAS) and transcriptome analysis are key methods for identifying genes linked to stress tolerance. GWAS helps identify genetic markers associated with stress resilience by examining genetic variations and phenotypic data across plant populations [69,79]. Transcriptome analysis offers a comprehensive view of gene expression in response to specific stress conditions, helping to uncover and validate genes that exhibit significant changes in expression related to stress tolerance [69,79].

Once the relevant genes are identified, gene editing technologies can be employed to either upregulate or downregulate their expression to enhance crop stress tolerance. The CRISPR/Cas9 system can be used to insert these genes into the crop genome or to perform targeted editing of existing homologous genes. For example, CRISPR/Cas9-mediated knockout of the *OsGS3* gene in rice and the *ZmGS3* gene in corn has been shown to improve tolerance to alkali stress [69]. Similarly, knocking out the *OsbHLH024* gene in rice with CRISPR/Cas9 enhances the expression of ion transport proteins, boosting the plant’s salt tolerance [112]. Additionally, replacing the natural *ARGOS8* promoter in corn with the *GOS2* promoter using CRISPR/Cas9 increases corn yield under drought conditions [113].

Genetic engineering techniques, particularly CRISPR/Cas9 genome editing, when combined with genome-wide association studies and transcriptome analysis, offer the potential to not only enhance the expression of alkali-tolerance genes in plants, but also to develop new alkali-resistant crop varieties. These innovations are important for tackling the global challenge of alkaline soils. It not only improves crop yield and quality but can also contribute to sustainable agriculture.

### 4.3. Crop Management Practices

Agricultural management practices play a crucial role in mitigating the negative impact of alkali stress on plant growth. The appropriate use of gypsum (calcium sulfate) or sulfuric acid can help lower the soil pH and dissolve carbonate compounds, thereby reducing alkalinity [114]. Some organic amendments, including compost and manure, not only improve soil structure and water retention capacity but can also promote beneficial microbial communities [115]. These microbes can enhance nutrient availability and stress tolerance.

## 5. Conclusions and Future Perspectives

The above analysis discusses the adverse impacts of alkaline stress on plant growth and development, as well as the self-defensive mechanisms involved to cope with alkaline stress in plants. To ensure their normal growth, plants have developed a variety of defense mechanisms to deal with alkaline stress, which can help them adapt to alkaline environments by reducing oxidative damage and maintaining cellular homeostasis. For example, hormones such as auxins [53,54,55,56], abscisic acid [57,59,116], and jasmonic acid [62,63] can enhance plant alkali tolerance by regulating antioxidant enzymatic activities, osmotic substances, and chlorophyll content. Scavenging reactive oxygen species is the primary strategy for plants to reduce oxidative damage and enhance their antioxidant system [36,64,67]. A high pH environment due to alkaline stress exacerbates adverse effects induced by ion imbalance. In response, plants activate a series of ion channels and transporters, such as various ion channels in the SOS pathway and hydrogen ion pumps, to regulate the outflow of sodium and hydrogen ions, thus, sustaining the internal and external ion balance of cells and protecting cells from alkaline stress damage [76,79,81]. Additionally, the specific expression of alkaline stress-related genes [94,95,96] and transcription factors [88,91] can also enhance plant alkali tolerance.

Soil alkalization is a global issue that endangers crop productivity. Soil microorganisms that can enhance plant alkalinity tolerance, such as *B. licheniformis* Jrh14–10 [99], *Enterobacter* sp. FN0603 [102], and how to improve plant alkali tolerance through microbial management can be explored in the future. Simultaneously, significant development has been made in understanding the molecular mechanisms of plant responses to saline-alkali stress. Our knowledge of alkali stress remains incomplete, particularly regarding how plants sense it. Scientists have identified sensors for osmotic stress (OSCA1 [117]), Na^+^ (MOCA1 [118]), and H_2_O_2_ (HPCA1 [119]) using forward genetic screening methods, but the sensor for perceiving alkali stress is still unknown. Identifying the alkali stress sensor and elucidating how plants perceive changes in external pH can enhance our understanding of the molecular mechanisms of plant responses to alkali stress, thereby addressing the complex challenges posed by alkali stress and promoting sustainable agricultural development. Therefore, it is highly worthwhile to explore the use of forward genetic screening methods to screen for alkali stress sensors.

Integrating multi-omics, genetic engineering, and advanced biotechnologies can offer a deeper understanding of plant alkali tolerance mechanisms, tackle the challenges posed by alkali stress and contribute to sustainable agriculture production.

## Figures and Tables

**Table 1:** Overview of physiological and molecular responses in different plants under alkaline stress conditions.

Plants	Alkaline Stress	Testing Index	Refs.
*Brassica rapa*	25 mM CaCO_3_ pH 7.5–8.0.	Biomass, nutrient accumulation, oxidative stress, photosynthesis parameters	[97]
Cotton (*Gossypium hirsutum*)	50 mM Na_2_CO_3_ pH 11.11	Anthocyanin and cyanidin content, germination potential, POD activity	[66]
*Arabidopsis*, soybean (*Glycine soja*)	50 mM NaHCO_3_	Growth parameters, expression of related genes	[94]
Soybean (*Glycine soja*)	50 mM NaHCO_3_	CAT activity, hydrogen peroxide(H_2_O_2_) content	[95]
Soybean (*Glycine soja*)	100 mM NaHCO_3_	Enzyme activity and kinetic characteristics, gene transcript level, growth parameters	[96]
Rice (*Oryza sativa*)	50 mM NaHCO_3_:Na_2_CO_3_ = 9:1 pH 9.17	Growth parameters, osmomodulatory substances, inorganic phosphorus, ATP and pyrophosphate, photosynthetic parameters, chlorophyll, sucrose and starch contents	[98]
*Nitraria tangutorum*	0, 10, 30, 50, 100, 150 mM NaHCO_3_:Na_2_CO_3_ = 9:1	Relative water, carbohydrates, flavonoid and anthocyanin secondary metabolites, antioxidant enzyme activity (CAT, SOD, GST, APX), electrical leakage, MDA, H_2_O_2,_ O_2_^−^, oxidative stress, Na^+^/K^+^ ratios, osmomodulatory substances, PI, stomatal aperture, intercellular CO_2_ concentration, chlorophyll	[14]
*Arabidopsis*	0, 75, 100, 150 mM NaHCO_3_	Growth parameters, bacteria quantity, Fv/Fm, non-photochemical quenching, photochemical quenching, MDA, oxidative stress, antioxidant enzyme activity (CAT, SOD, GST, APX, GPX, MDHAR, DHAR)	[99]
*Brassica napus* L.	2% NaCl, pH 9.0	Growth parameters, biomass, total antioxidant capacity levels, chlorophyll, bacteria quantity	[100]
*Hordeum bogdanii*	50,100 mM Na_2_CO_3_:NaHCO_3_ = 1:1	Expression levels of gene *HbNHX1*, Na^+^, K^+^, Cl^−^, SO_4_^2−^, NO^3−^ contents	[101]
Wheat (*Triticum aestivum*)	100 mM NaCl, pH 9.0	Growth parameters, chlorophyll, soil enzyme activity, carotenoid content, osmomodulatory substances, antioxidant enzyme activity (CAT, SOD, POD)	[102]
Strawberry	40 mM NaHCO_3_	Gas exchange parameters, PI, Fv, Fm, RE0/RC, yield, DW, chlorophyll a fluorescence induction curves, chlorophyll and carotenoid content	[103]
Sugar beet (*Beta vulgaris* L.)	15 mM–100 mM NaHCO_3_	FW and DW of shoots and roots, expression levels of nine selected genes	[104]
*Sesbania cannabina*	Saline-alkaline field (pH 9.7) black soil (pH 7.2)	Conductivity, soil organic carbon	[105]
Rice (*Oryza sativa* L.)	25 mM NaCO_3_, pH10.0	Seed vigor, LOX10 activity assay on α-linoleic acid, MDA, chlorophyll, expression of related genes, linolenic acid and linoleic acid concentrations	[106]
*Trollius chinensis*	40, 80, 120, 160 mM NaCl:Na_2_SO_4_:NaHCO_3_: Na_2_CO_3_ = 1:9:9:1	Leaf RWC, osmomodulatory substances, MDA, H_2_O_2,_ O_2_^•–^, SOD and POD activity, AsA and GSH content, APX and GR activity	[107]
*Leymus chinensis*	Saline-alkali soil pH 8–10	Soil properties and vegetation features, variation of NO_2_^−^-N concentration, inorganic nitrogen concentration in rhizosphere and non-rhizosphere soil, bacteria quantity, biomass, root exudates mass in medium, residual inorganic nitrogen, pH in the medium	[87]

Growth parameters including root/shoot length, fresh weight (FW), dry weight (DW), grain weight, germination rate. POD: peroxidase; GST: glutathione S-transferase; APX: ascorbate peroxidase; GPX: glutathione peroxidase; MDHAR: monodehydroascorbate reductase; DHAR: dehydroascorbate reductase; AsA: ascorbic acid; APX: ascorbate peroxidase; GR: glutathione reductase; PI: performance index; Fv: variable fluorescence; Fm: maximum fluorescence; RE0/RC: regulation energy 0/relative chlorophyll content; RWC: relative water content.

## References

[1] Xv Z.Q., Xia G.M. (2018). Research progress on the causes, characteristics and remediation measures of soda saline-alkali soil in the songnen plain. Soil Water Conserv. China.

[2] Wang S.J., Chen Q., Li Y., Zhuo Y.Q., Xu L.Z. (2017). Research on saline-alkali soil amelioration with FGD gypsum. Resour. Conserv. Recycl..

[3] Pearce F. (2014). The $27 billion cost of salt poisoning. New Sci..

[4] Guo S.S., Ruan B.Q., Chen H.R., Guan X.Y., Wang S.L., Xu N.N., Li Y.P. (2018). Characterizing the spatiotemporal evolution of soil salinization in Hetao Irrigation District (China) using a remote sensing approach. Int. J. Remote Sens..

[5] Yang C.W., Shi D.C., Wang D.L. (2008). Comparative effects of salt and alkali stresses on growth, osmotic adjustment and ionic balance of an alkali-resistant halophyte *Suaeda glauca* (Bge.). Plant Growth Regul..

[6] Shi D.C., Wang D.L. (2005). Effects of various salt-alkaline mixed stresses on *Aneurolepidium chinense* (Trin.) Kitag. Plant Soil.

[7] Cosgrove D.J. (2000). Loosening of plant cell walls by expansins. Nature.

[8] Bibikova T.N., Jacob T., Dahse I., Gilroy S. (1998). Localized changes in apoplastic and cytoplasmic pH are associated with root hair development in *Arabidopsis thaliana*. Development.

[9] Agathokleous E., Feng Z.Z., Peñuelas J. (2020). Chlorophyll hormesis: Are chlorophylls major components of stress biology in higher plants?. Sci. Total Environ..

[10] Liu H., Wang Z., Dong S., Zou J.X., Jin H. (2022). Mitigation effect of exogenous 5 -aminolevulinic acid on populus wutunensis seedling under saline-alkali stress. J. N. East For. Univ..

[11] Li X.Z., Wang S., Chen X.J., Cong Y.D., Cui J.X., Shi Q.H., Liu H.Y., Diao M. (2022). The positive effects of exogenous sodium nitroprusside on the plant growth, photosystem II efficiency and Calvin cycle of tomato seedlings under salt stress. Sci. Hortic..

[12] Soliman W.S., Fujimori M., Tase K., Sugiyama S. (2011). Oxidative stress and physiological damage under prolonged heat stress in C_3_ grass *Lolium perenne*. Grassl. Sci..

[13] Soliman W.S., Fujimori M., Tase K., Sugiyama S. (2012). Heat tolerance and suppression of oxidative stress: Comparative analysis of 25 cultivars of the C_3_ grass *Lolium perenne*. Environ. Exp. Bot..

[14] Zhang J., Cheng K., Liu X., Dai Z., Zheng L., Wang Y. (2023). Exogenous abscisic acid and sodium nitroprusside regulate flavonoid biosynthesis and photosynthesis of Nitraria tangutorum Bobr in alkali stress. Front. Plant Sci..

[15] Xv F.F., Luo Y.Q. (2012). The impact of mixed saline-alkali stress on the germination of rice seeds. Seed.

[16] Li H.Y., Pan S.J., Qian Y.D., Ma Y., Si Y., Gao S., Zheng G.P., Jiang Y.W., Zhou J. (2015). The impact of mixed saline-alkali stress on the yield and quality of cold-region rice. J. South Agric..

[17] He Q., Yang F., Feng W.D., Ma H.W., Zhang W.Y., Yin Y.B., Wang X. (2021). A comparative study on the impact of saline-alkali stress on the quality of rice in Ningxia. J. Ningxia Agric. For. Sci. Technol..

[18] Weng Y.J. (2003). Crop Salt-Tolerant Varieties and Their Cultivation Techniques.

[19] Yv T.Y., Wang C.X., Sun X.W., Sun X.S., Zheng Y.M., Wu Z.F., Shen P., Wu C.B. (2017). The impact of alkaline stress on the root morphology and dry matter accumulation of peanut seedlings. Chin. J. Oil Crop Sci..

[20] Shi D.C., Sheng Y.M. (2005). Effect of various salt-alkaline mixed stress conditions on sunflower seedlings and analysis of their stress factors. Environ. Exp. Bot..

[21] Marty F. (1999). Plant vacuoles. Plant Cell.

[22] Felle H.H. (2001). pH: Signal and messenger in plant cells. Plant Biol..

[23] Chen S.X., Li L., Jiao X.Z. (1998). The impact of osmotic stress on protein phosphorylation in Dunaliella salina cells. J. Integr. Plant Biol..

[24] Jing C.L., Xu Z.C., Zou P., Tang Q., Li Y.Q., You X.W., Zhang C.S. (2019). Coastal halophytes alter properties and microbial community structure of the saline soils in the Yellow River Delta, China. Appl. Soil Ecol..

[25] Azadi N., Raiesi F. (2021). Salinization depresses soil enzyme activity in metal-polluted soils through increases in metal mobilization and decreases in microbial biomass. Ecotoxicology.

[26] Cao D., Shi F.C., Koike T., Lu Z.H., Sun J.K. (2014). Halophyte plant communities affecting enzyme activity and microbes in saline soils of the yellow river delta in China. Clean-Soil Air Water.

[27] Zhang T., Wan S., Kang Y., Feng H. (2014). Urease activity and its relationships to soil physiochemical properties in a highly saline-sodic soil. J. Soil Sci. Plant Nutr..

[28] Yang W.G., Han Y., Zheng F.L., Wang Z.L., Yi Y., Feng Z.Z. (2016). Investigating spatial distribution of soil quality index and its impacts on corn yield in a cultivated catchment of the Chinese mollisol region. Soil Sci. Soc. Am. J..

[29] Hasegawa P.M. (2013). Sodium (Na^+^) homeostasis and salt tolerance of plants. Environ. Exp. Bot..

[30] Liu D., Cong R.C., Dang H.Z., Li Q.M., Liu D.X., Yang Q.S. (2014). Comparative effects of salt and alkali stresses on plant physiology of willow. Ecol. Environ. Sci..

[31] Qin Y., Bai J.H., Wang Y.Q., Liu J.H., Hui Y.N., Dong Z., Ji L. (2018). Comparative effects of salt and alkali stress on photosynthesis and root physiology of oat at anthesis. Arch. Biol. Sci..

[32] Guo R., Li F., Zhou J., Li H.R., Xia X., Liu Q. (2016). Eco-physiological responses of linseed (*Linum usitatissimum*) to salt and alkali stresses. Chin. J. Plant Ecol..

[33] Shao S., Li M.X., Yang D.S., Zhang J., Shi L.X. (2016). The physiological variations of asaptation mechaniam in *Glycine soja* seedlings under saline and alkaline stresses. Pak. J. Bot..

[34] Yang Y., Wu Y., Ma L., Yang Z., Dong Q., Li Q., Ni X., Kudla J., Song C., Guo Y. (2019). The Ca^2+^ sensor SCaBP3/CBL7 modulates plasma membrane H^+^-ATPase activity and promotes alkali tolerance in *Arabidopsis*. Plant Cell.

[35] Zhao H.Y., Lin H.X. (2020). Molecular mechanism of plants in responses to salt and alkali stress. Soils Crops.

[36] Apel K., Hirt H. (2004). Reactive oxygen species: Metabolism, oxidative stress, and signal transduction. Annu. Rev. Plant Biol..

[37] Maurya A.K. (2020). Oxidative Stress in Crop Plants.

[38] Berlett B.S., Stadtman E.R. (1997). Protein oxidation in aging, disease, and oxidative stress. J. Biol. Chem..

[39] Zhang Y.F., Yin B. (2009). Influences of salt and alkali mixed stresses on antioxidative activity and MDA content of Medicago sativa at seedling stage. Acta Pratacult. Sin..

[40] Juan C.A., Pérez de la Lastra J.M., Plou F.J., Pérez-Lebeña E. (2021). The Chemistry of Reactive Oxygen Species (ROS) Revisited: Outlining Their Role in Biological Macromolecules (DNA, Lipids and Proteins) and Induced Pathologies. Int. J. Mol. Sci..

[41] Kosová K., Vítámvás P., Prášil I.T., Klíma M., Renaut J. (2021). Plant Proteoforms Under Environmental Stress: Functional Proteins Arising From a Single Gene. Front. Plant Sci..

[42] Guo R., Zhou Z.Y., Cai R., Liu L., Wang R.X., Sun Y.G., Wang D., Yan Z., Guo C.H. (2024). Metabolomic and physiological analysis of alfalfa (*Medicago sativa* L.) in response to saline and alkaline stress. Plant Physiol. Biochem..

[43] Carillo P., Annunziata M.G., Pontecorvo G., Fuggi A., Woodrow P. (2011). Salinity Stress and Salt Tolerance. Abiotic Stress Plants-Mech. Adapt..

[44] Guo R., Zhou J., Ren G.X., Hao W.P. (2013). Physiological responses of linseed seedlings to iso osmotic polyethylene glycol, salt, and alkali stresses. Agron. J..

[45] Wang W., Pang J., Zhang F., Sun L., Yang L., Zhao Y., Yang Y., Wang Y., Siddique K.H.M. (2021). Integrated transcriptomics and metabolomics analysis to characterize alkali stress responses in canola (*Brassica napus* L.). Plant Physiol. Biochem..

[46] Jain M., Mathur G., Koul S., Sarin N. (2001). Ameliorative effects of proline on salt stress-induced lipid peroxidation in cell lines of groundnut (*Arachis hypogaea* L.). Plant Cell Rep..

[47] Liang X.J., Wang Y.J., Li Y.K., An W., He X.R., Chen Y.Z., Shi Z.G., He J., Wan R. (2022). Widely-targeted etabolic profiling in *Lycium barbarum* fruits under salt-alkaline stress uncovers mechanism of salinity tolerance. Molecules.

[48] Zhu Y.F., Jia X.M., Wu Y.X., Hu Y., Cheng L., Zhao T., Huang Z.C., Wang Y.X. (2020). Quantitative proteomic analysis of *Malus halliana* exposed to salt-alkali mixed stress reveals alterations in energy metabolism and stress regulation. Plant Growth Regul..

[49] Ma Q., Wang H.L., Wu E.G., Zhang H., Feng Y., Feng B.L. (2023). Widely targeted metabolomic analysis revealed the effects of alkaline stress on nonvolatile and volatile metabolites in broomcorn millet grains. Food Res. Int..

[50] Jiao Y., Bai Z.Z., Xu J.Y., Zhao M.L., Khan Y., Hu Y.J., Shi L.X. (2018). Metabolomics and its physiological regulation process reveal the salt-tolerant mechanism in *Glycine soja* seedling roots. Plant Physiol. Biochem..

[51] Lv X.L., Zhu L., Ma D.M., Zhang F.J., Cai Z.Y., Bai H.B., Hui J., Li S.H., Xu X., Li M. (2024). Integrated metabolomics and transcriptomics analyses highlight the flavonoid compounds response to alkaline salt stress in *Glycyrrhiza uralensis* Leaves. J. Agric. Food Chem..

[52] Wu J.X., Cao J.Y., Su M., Feng G.Z., Xu Y.H., Yi H.L. (2019). Genome-wide comprehensive analysis of transcriptomes and small RNAs offers insights into the molecular mechanism of alkaline stress tolerance in a citrus rootstock. Hortic. Res..

[53] He Y., Zhang T., Sun Y., Wang X.J., Cao Q.Q., Fang Z.G., Chang M., Cai Q.S., Lou L.Q. (2022). Exogenous IAA alleviates arsenic toxicity to rice and reduces arsenic accumulation in rice grains. J. Plant Growth Regul..

[54] Gong B.A., Miao L., Kong W.J., Bai J.G., Wang X.F., Wei M., Shi Q.H. (2014). Nitric oxide, as a downstream signal, plays vital role in auxin induced cucumber tolerance to sodic alkaline stress. Plant Physiol. Biochem..

[55] Ma C.K., Yuan S., Xie B., Li Q., Wang Q.J., Shao M.A. (2022). IAA plays an important role in alkaline stress tolerance by modulating root development and ROS detoxifying systems in rice plants. Int. J. Mol. Sci..

[56] Xu W., Jia L., Baluška F., Ding G., Shi W., Ye N., Zhang J. (2012). PIN2 is required for the adaptation of *Arabidopsis* roots to alkaline stress by modulating proton secretion. J. Exp. Bot..

[57] Liu X.L., Zhang H., Jin Y.Y., Wang M.M., Yang H.Y., Ma H.Y., Jiang C.J., Liang Z.W. (2019). Abscisic acid primes rice seedlings for enhanced tolerance to alkaline stress by upregulating antioxidant defense and stress tolerance-related genes. Plant Soil.

[58] Liu X.L., Xie X.Z., Zheng C.K., Wei L.X., Li X.W., Jin Y.Y., Zhang G.H., Jiang C.J., Liang Z.W. (2022). RNAi-mediated suppression of the abscisic acid catabolism gene *OsABA8ox1* increases abscisic acid content and tolerance to saline-alkaline stress in rice (*Oryza sativa* L.). Crop J..

[59] Xu Z.J., Wang J.C., Zhen W.T., Sun T., Hu X.H. (2022). Abscisic acid alleviates harmful effect of saline-alkaline stress on tomato seedlings. Plant Physiol. Biochem..

[60] Li B., Wu T.T., Fang Z.J., Li H., Yang Z., Lin H. (2019). The impact of abscisic acid on mineral element content in nutritive organs of alfalfa seedlings under mixed soda salt-alkaline stress. Acta Agrestia Sin..

[61] Wei L.X., Lv B.S., Wang M.M., Ma H.Y., Yang H.Y., Liu X.L., Jiang C.J., Liang Z.W. (2015). Priming effect of abscisic acid on alkaline stress tolerance in rice (*Oryza sativa* L.) seedlings. Plant Physiol. Biochem..

[62] Mir M.A., John R., Alyemeni M.N., Alam P., Ahmad P. (2018). Jasmonic acid ameliorates alkaline stress by improving growth performance, ascorbate glutathione cycle and glyoxylase system in maize seedlings. Sci. Rep..

[63] Zhu D., Cai H., Luo X., Bai X., Deyholos M.K., Chen Q., Chen C., Ji W., Zhu Y.M. (2012). Over-expression of a novel JAZ family gene from *Glycine soja*, increases salt and alkali stress tolerance. Biochem. Biophys. Res. Commun..

[64] Lu X., Min W., Shi Y., Tian L., Li P., Ma T., Zhang Y., Luo C. (2022). Exogenous melatonin alleviates alkaline stress by removing reactive oxygen species and promoting antioxidant defence in rice seedlings. Front. Plant Sci..

[65] Zhang H., Liu X.L., Zhang R.X., Yuan H.Y., Wang M.M., Yang H.Y., Ma H.Y., Liu D., Jiang C.J., Liang Z.W. (2017). Root damage under alkaline stress is associated with reactive oxygen species accumulation in rice (*Oryza sativa* L.). Front. Plant Sci..

[66] Jiang T.T., He Y.X., Wu Z., Cui Y.P., Wang X.P., Huang H., Fan Y.P., Han M.G., Wang J.J., Wang S. (2023). Enhancing stimulation of cyaniding, *GhLDOX3* activates reactive oxygen species to regulate tolerance of alkalinity negatively in cotton. Ecotoxicol. Environ. Saf..

[67] Sun N., Song T.T., Ma Z.Y., Dong L., Zhan L.F., Xing Y.M., Liu J.M., Song J.X., Wang S., Cai H. (2020). Overexpression of *MsSiR* enhances alkali tolerance in alfalfa (*Medicago sativa* L.) by increasing the glutathione content. Plant Physiol. Biochem..

[68] Sun X.L., Sun M.Z., Jia B.W., Qin Z.W., Yang K.J., Chen C., Yu Q.Y., Zhu Y.M. (2016). A *Glycine soja* methionine sulfoxide reductase B5a interacts with the Ca^2+^/CAM-binding kinase GsCBRLK and activates ROS signaling under carbonate alkaline stress. Plant J..

[69] Zhang H.L., Yu F.F., Xie P., Sun S.Y., Qiao X.H., Tang S.Y., Chen C.X., Yang S., Mei C., Yang D.K. (2023). A Gγ protein regulates alkaline sensitivity in crops. Science.

[70] Latef A.A.A., Tran L.S.P. (2016). Impacts of priming with silicon on the growth and tolerance of maize plants to alkaline stress. Front. Plant Sci..

[71] Li J., Yang Y. (2023). How do plants maintain pH and ion homeostasis under saline-alkali stress?. Front. Plant Sci..

[72] Hussain S., Ali B., Ren X.L., Chen X.L., Li Q.Q., Saqib M., Ahmad N. (2021). Recent progress in understanding salinity tolerance in plants: Story of Na^+^/K^+^ balance and beyond. Plant Physiol. Biochem..

[73] Cao Y., Song H., Zhang L. (2022). New Insight into Plant Saline-Alkali Tolerance Mechanisms and Application to Breeding. Int. J. Mol. Sci..

[74] Waheed A., Zhuo L., Wang M.H., Hailiang X., Tong Z.W., Wang C.H., Aili A. (2024). Integrative mechanisms of plant salt tolerance: Biological pathways, phytohormonal regulation, and technological innovations. Plant Stress.

[75] Rao Y., Peng T., Xue S.W. (2023). Mechanisms of plant saline-alkaline tolerance. J. Plant Physiol..

[76] Ji H.T., Pardo J.M., Batelli G.G., Van Oosten M.J., Bressan R.A., Li X. (2013). The salt overly sensitive (SOS) pathway: Established and emerging roles. Mol. Plant.

[77] Shen T., Yan R.J., Xu F.J., Wang Q.W., Chen D., Li K.Y., Ni L., Jiang M.Y. (2023). The NADPH oxidase OsRbohD and OsRbohH negatively regulate saline-alkaline tolerance in rice. Environ. Exp. Bot..

[78] Gong B., Wen D., Wang X., Wei M., Yang F., Li Y., Shi Q. (2015). S-nitrosoglutathione reductase-modulated redox signaling controls sodic alkaline stress responses in *Solanum lycopersicum* L.. Plant Cell Physiol..

[79] Cao Y.B., Zhang M., Liang X.Y., Li F.R., Shi Y.L., Yang X.H., Jiang C.F. (2020). Natural variation of an EF-hand Ca^2+^-binding-protein coding gene confers saline-alkaline tolerance in maize. Nat. Commun..

[80] Li M.T., Guo P., Nan N., Ma A., Liu W.X., Wang T.J., Yun D.J., Xu Z.Y. (2024). Plasma membrane-localized H^+^-ATPase OsAHA3 functions in saline-alkaline stress tolerance in rice. Plant Cell Rep..

[81] Fuglsang A.T., Guo Y., Cuin T.A., Qiu Q.S., Song C.P., Kristiansen K.A., Bych K., Schulz A., Shabala S., Schumaker K.S. (2007). *Arabidopsis* protein kinase PKS5 inhibits the plasma membrane H^+^-ATPase by preventing interaction with 14-3-3 protein. Plant Cell.

[82] Yang Y.Q., Qin Y.X., Xie C.G., Zhao F.Y., Zhao J.F., Liu D.F., Chen S.Y., Fuglsang A.T., Palmgren M.G., Schumaker K.S. (2010). The *Arabidopsis* chaperone J3 regulates the plasma membrane H^+^-ATPase through interaction with the PKS5 kinase. Plant Cell.

[83] Li J.S., Yang H.B., Peer W.A., Richter G., Blakeslee J., Bandyopadhyay A., Titapiwantakun B., Undurraga S., Khodakovskaya M., Richards E.L. (2005). *Arabidopsis* H^+^-PPase AVP1 regulates auxin-mediated organ development. Science.

[84] Park S., Li J.S., Pittman J.K., Berkowitz G.A., Yang H.B., Undurraga S., Morris J., Hirschi K.D., Gaxiola R.A. (2005). Up-regulation of a H^+^-pyrophosphatase (H^+^-PPase) as a strategy to engineer drought-resistant crop plants. Proc. Natl. Acad. Sci. USA.

[85] Song C.P., Guo Y., Qiu Q.S., Lambert G., Galbraith D.W., Jagendorf A., Zhu J.K. (2004). A probable Na^+^(K^+^)/H^+^ exchanger on the chloroplast envelope functions in pH homeostasis and chloroplast development in *Arabidopsis thaliana*. Proc. Natl. Acad. Sci. USA.

[86] Yamaguchi T., Aharon G.S., Sottosanto J.B., Blumwald E. (2005). Vacuolar Na^+^/H^+^ antiporter cation selectivity is regulated by calmodulin from within the vacuole in a Ca^2^ and pH dependent manner. Proc. Natl. Acad. Sci. USA.

[87] Wang G., Zhang L.H., Guo Z.H., Shi D.F., Zhai H.L., Yao Y., Yang T.X., Xin S.Q., Cui H.Y., Li J.Q. (2023). Benefits of biological nitrification inhibition of *Leymus chinensis* under alkaline stress: The regulatory function of ammonium-N exceeds its nutritional function. Front. Plant Sci..

[88] Mu Y.J., Zhan Y.J., Xv W.F., Xia T.Y. (2020). Transcriptional and network responses of *Arabidopsis* roots under high pH stress. Acta Pedol. Sin..

[89] Liu T.F., Wang Y., Li X.F., Che H.T., Zhang Y.N. (2024). *LpNAC5* positively regulates drought, salt and alkalinity tolerance of *Lilium pumilum*. Gene.

[90] Yu Y., Duan X., Ding X., Chen C., Zhu D., Yin K., Cao L., Song X., Zhu P., Li Q. (2017). A novel AP2/ERF family transcription factor from *Glycine soja*, GsERF71, is a DNA binding protein that positively regulates alkaline stress tolerance in *Arabidopsis*. Plant Mol. Biol..

[91] Zhu D., Li R.T., Liu X., Sun M.Z., Wu J., Zhang N., Zhu Y.M. (2014). The positive regulatory roles of the TIFY10 Proteins in plant responses to alkaline stress. PLoS ONE.

[92] Zhao C.Y., Pan X.W., Yu Y., Zhu Y.M., Kong F.J., Sun X., Wang F.F. (2020). Overexpression of a TIFY family gene, *GsJAZ2*, exhibits enhanced tolerance to alkaline stress in soybean. Mol. Breed..

[93] Liu Z.J., Hu Y.Z., Du A.P., Yu L., Fu X.Y., Wu C.L., Lu L.X., Liu Y.X., Wang S.H., Huang W.Z. (2022). Cell Wall Matrix Polysaccharides Contribute to Salt-Alkali Tolerance in Rice. Int. J. Mol. Sci..

[94] Ding D.Q., Mi X., Wu J.Y., Nisa Z.U., Elansary H.O., Jin X.X., Yu L.J., Chen C. (2023). GsPKS24, a calcineurin B-like protein-interacting protein kinase gene from *Glycine soja*, positively regulates tolerance to pH stress and ABA signal transduction. Funct. Integr. Genom..

[95] Lu P., Dai S.Y., Yong L.T., Zhou B.H., Wang N., Dong Y.Y., Liu W.C., Wang F.W., Yang H.Y., Li X.W. (2023). A soybean sucrose non-fermenting protein kinase 1 Gene, *GmSNF1*, positively regulates plant response to salt and salt-alkali stress in transgenic plants. Int. J. Mol. Sci..

[96] Jiao F., Chen Y., Zhang D.D., Wu J.H. (2023). Genome-wide characterization of soybean hexokinase genes reveals a positive role of *GmHXK15* in alkali stress response. Plants.

[97] Navarro-León E., Grazioso A., Atero-Calvo S., Esposito S., Blasco B., Rios J.J. (2023). Evaluation of the alkalinity stress tolerance of three Brassica rapa CAX1 TILLING mutants. Plant Physiol. Biochem..

[98] Wang B., Xie G.Q., Liu Z.L., He R., Han J., Huang S.C., Liu L.H., Cheng X.G. (2019). Mutagenesis Reveals That the *OsPPa6* Gene Is Required for Enhancing the Alkaline Tolerance in Rice. Front. Plant Sci..

[99] Guo L.F., Zhang X.C., Zhao J.W., Zhang A.Q., Pang Q.Y. (2023). Enhancement of Sulfur Metabolism and Antioxidant Machinery Confers *Bacillus* sp. Jrh14-10–induced Alkaline Stress Tolerance in Plant. Plant Physiol. Biochem..

[100] Zhuang X.L., Liu Y., Fang N., Bai Z.H., Gao J. (2023). Quorum sensing improves the plant growth-promoting ability of Stenotrophomonas rhizophila under saline-alkaline stress by enhancing its environmental adaptability. Front. Microbiol..

[101] Long F., Hu M.F., Chen S., Bao G.S., Dan H., Chen S.H. (2023). Endophytic Fungi Regulate *HbNHX1* Expression and Ion Balance in *Hordeum bogdanii* under Alkaline Stress. J. Fungi.

[102] Xu F., Liang Y., Wang X., Guo Y., Tang K., Feng F. (2022). Synergic mitigation of saline-alkaline stress in wheat plant by silicon and *Enterobacter* sp. FN0603. Front. Microbiol..

[103] Malekzadeh M.R., Roosta H.R., Kalaji H.M. (2023). GO nanoparticles mitigate the negative effects of salt and alkalinity stress by enhancing gas exchange and photosynthetic efficiency of strawberry plants. Sci. Rep..

[104] Wu G.Q., Li Z.Q., Cao H., Wang J.L. (2019). Genome-wide identification and expression analysis of the *WRKY* genes in sugar beet (*Beta vulgaris* L.) under alkaline stress. PeerJ.

[105] Luo H.F., Wang X.F., You C.Q., Wu X.D., Pan D.F., Lv Z.Y., Li T., Zhang D.M., Shen Z.B., Zhang X.D. (2024). Telomere-to-telomere genome of the allotetraploid legume *Sesbania cannabina* reveals transposon-driven subgenome divergence and mechanisms of alkaline stress tolerance. Sci. China-Life Sci..

[106] Wang F., Xu H., Zhang L., Shi Y., Song Y., Wang X., Cai Q., He W., Xie H., Zhang J. (2023). The lipoxygenase OsLOX10 affects seed longevity and resistance to saline-alkaline stress during rice seedlings. Plant Mol. Biol..

[107] Hou R.W., Yang L.Z., Wuyun T., Chen S.Y., Zhang L. (2023). Genes related to osmoregulation and antioxidation play important roles in the response of *Trollius chinensis* seedlings to saline-alkali stress. Front. Plant Sci..

[108] Deng H.Z., Wang C.H., Xv Q., Yuan X.P., FEng Y., Yu H.Y., Wang Y.P., Wei X.H. (2015). A comparative analysis of genetic diversity between Chinese Indigenous and cultivated rice varieties. J. Plant Genet. Resour..

[109] Guo Y., Hua Q.C., Hu M.X., Wang Y., Yuan J.J., Yang F.P. (2023). The application and prospects of molecular marker-assisted selection in crop breeding. J. Cold-Arid Agric. Sci..

[110] Ma X., Zhang Q., Zhu Q., Liu W., Chen Y., Qiu R., Wang B., Yang Z., Li H., Lin Y. (2015). A robust CRISPR/Cas9 system for convenient, high-efficiency multiplex genome editing in monocot and dicot plants. Mol. Plant.

[111] Zhao S.S., Di Y.H., Hao G.F. (2019). Research progress of CRISPR-Cas9 gene editing technology in gene function and crop breeding. Mol. Plant Breed..

[112] Alam M.S., Kong J., Tao R., Ahmed T., Alamin M., Alotaibi S.S., Abdelsalam N.R., Xu J.H. (2022). CRISPR/Cas9 mediated knockout of the *OsbHLH024* transcription factor improves salt stress resistance in rice (*Oryza sativa* L.). Plants.

[113] Shi J.R., Gao H.R., Wang H.Y., Lafitte H.R., Archibald R.L., Yang M.Z., Hakimi S.M., Mo H., Habben J.E. (2017). *ARGOS8* variants generated by CRISPR-Cas9 improve maize grain yield under field drought stress conditions. Plant Biotechnol. J..

[114] Sun J.J., Ma B., Li F.J., Han F., Yao G.D., WAng Y.G. (2024). Effects of applying flue gas desulfurized gypsum on improvement and carbon sequestration in saline-sodic soils. China Powder Sci. Technol..

[115] Liu X., Liu H., Zhang Y., Liu C., Liu Y., Li Z., Zhang M. (2023). Organic amendments alter microbiota assembly to stimulate soil metabolism for improving soil quality in wheat-maize rotation system. J. Environ. Manag..

[116] Wei T.J., Wang M.M., Jin Y.Y., Zhang G.H., Liu M., Yang H.Y., Jiang C.J., Liang Z.W. (2021). Abscisic acid priming creates alkaline tolerance in alfalfa seedlings (*Medicago sativa* L.). Agriculture.

[117] Yuan F., Yang H., Xue Y., Kong D., Ye R., Li C., Zhang J., Theprungsirikul L., Shrift T., Krichilsky B. (2014). OSCA1 mediates osmotic-stress-evoked Ca^2+^ increases vital for osmosensing in Arabidopsis. Nature.

[118] Jiang Z., Zhou X., Tao M., Yuan F., Liu L., Wu F., Wu X., Xiang Y., Niu Y., Liu F. (2019). Plant cell-surface GIPC sphingolipids sense salt to trigger Ca^2+^ influx. Nature.

[119] Wu F., Chi Y., Jiang Z., Xu Y., Xie L., Huang F., Wan D., Ni J., Yuan F., Wu X. (2020). Hydrogen peroxide sensor HPCA1 is an LRR receptor kinase in *Arabidopsis*. Nature.

